# Ovariectomy reduces cholinergic modulation of excitatory synaptic transmission in the rat entorhinal cortex

**DOI:** 10.1371/journal.pone.0271131

**Published:** 2022-08-08

**Authors:** Ariel A. Batallán Burrowes, Olayemi Joseph Olajide, Isabella A. Iasenza, Waqqas M. Shams, Francis Carter, C. Andrew Chapman

**Affiliations:** 1 Department of Psychology, Center for Studies in Behavioral Neurobiology, Concordia University, Montréal, Québec, Canada; 2 Department of Anatomy, Division of Neurobiology, University of Ilorin, PMB, Ilorin, Nigeria; CNRS - ENS Paris Saclay, FRANCE

## Abstract

Estrogens are thought to contribute to cognitive function in part by promoting the function of basal forebrain cholinergic neurons that project to the hippocampus and cortical regions including the entorhinal cortex. Reductions in estrogens may alter cognition by reducing the function of cholinergic inputs to both the hippocampus and entorhinal cortex. In the present study, we assessed the effects of ovariectomy on proteins associated with cholinergic synapses in the entorhinal cortex. Ovariectomy was conducted at PD63, and tissue was obtained on PD84 to 89 to quantify changes in the degradative enzyme acetylcholinesterase, the vesicular acetylcholine transporter, and muscarinic M_1_ receptor protein. Although the vesicular acetylcholine transporter was unaffected, ovariectomy reduced both acetylcholinesterase and M_1_ receptor protein, and these reductions were prevented by chronic replacement of 17β-estradiol following ovariectomy. We also assessed the effects of ovariectomy on the cholinergic modulation of excitatory transmission, by comparing the effects of the acetylcholinesterase inhibitor eserine on evoked excitatory synaptic field potentials in brain slices obtained from intact rats, and from ovariectomized rats with or without 17β-estradiol replacement. Eserine is known to prolong the effects of endogenously released acetylcholine, resulting in an M_1_-like mediated reduction of glutamate release at excitatory synapses. The reduction in excitatory synaptic potentials in layer II of the entorhinal cortex induced by 15-min application of 10 μM eserine was greatly reduced in slices from ovariectomized rats as compared to intact rats and ovariectomized rats with replacement of 17β-estradiol. The reduced modulatory effect of eserine is consistent with the observed changes in cholinergic proteins, and suggests that reductions in 17β-estradiol following ovariectomy lead to impaired cholinergic function within the entorhinal cortex.

## Introduction

Estrogens are produced by the ovaries and synthesized in the brain, and are known to modulate cognitive functions in both humans and animals [1–3]. Cyclic increases of estrogens are associated with improved attention and recollection of verbal, emotional and spatial memory in humans [4–7] and rats show improved object recognition and working memory during proestrus when estrogens are high [8, 9]. Administration of estrogens also enhance working memory and hippocampal-dependent tasks including spatial navigation, novel object discrimination, and social learning [10, 11], and can promote the use of spatial cognitive strategies associated with the hippocampus [12–14].

Estrogens are thought to promote cognitive function by enhancing excitatory synaptic transmission though multiple mechanisms in the hippocampus and other cortical regions including the entorhinal cortex [15–18]. In the hippocampal CA1 region, 17β-estradiol (E2) reduces inhibitory synaptic transmission [19], facilitates excitatory transmission [20–22], and increases dendritic spine density [23]. We have also found that activation of G-protein estrogen receptor-1 (GPER1) receptors by E2 induces a rapid and reversible potentiation of excitatory synaptic responses in the entorhinal cortex [18], indicating that E2 can facilitate excitatory synaptic inputs to the entorhinal cortex. Estrogens are also thought to enhance cognition by promoting the function of basal forebrain cholinergic neurons. Basal forebrain cholinergic neurons contain estrogen receptor α [24, 25], and the transcription of choline acetyltransferase is modulated during the estrous cycle, and enhanced by E2 following ovariectomy [26, 27]. Cholinergic inputs to the hippocampus and entorhinal cortex promote neuronal synchronization and reduce excitatory transmission [28–30], and play central roles in attention, sensory processing, learning and memory, and spatial navigation [4, 15, 31–33].

Reductions in cholinergic function in the hippocampal region associated with a decline in circulating estrogens are thought to contribute to cognitive changes during the perimenopause transition [4]. Surgical removal of ovaries is associated with cognitive decline in women that is prevented by hormone replacement therapies containing estrogens [34, 35]. Ovariectomy in rats results in estrogen-dependent changes in cognitive function that are due in part to disruptions in cholinergic transmission [32, 36]. Ovariectomy results in estrogen-dependent reductions in the density of basal forebrain cholinergic neurons and reduces cholinergic terminals and acetylcholine release in the hippocampus [25, 37]. This can result in reduced activation of NMDA glutamate receptors and the impairment of hippocampal function [38, 39].

A reduction in cholinergic function following ovariectomy in the entorhinal cortex could have substantial effects on cognition by affecting both excitatory synaptic transmission and neuronal excitability [29]. Reduced cholinergic function could increase basal excitatory transmission by reducing the inhibition of glutamate release caused by acetylcholine [30], and could also reduce neuronal excitability because acetylcholine normally depolarizes the membrane potential of entorhinal neurons [40, 41]. In addition, the expression of theta- and gamma-frequency rhythmic population activities that contribute to mnemonic processing and spatial navigation are likely to be reduced because these rhythms are dependent on muscarinic depolarization of principle neurons and activation of GABA interneurons that synchronize population rhythms [29, 42].

To assess the effects of ovariectomy on cholinergic function in the entorhinal cortex, we measured the expression of proteins associated with cholinergic transmission three weeks following either a sham surgery, ovariectomy, or ovariectomy with chronic replacement of E2 [43, 44]. Entorhinal tissue samples were quantified for expression of the degradative enzyme acetylcholinesterase, the vesicular acetylcholine transporter (VAChT), and M_1_ muscarinic receptor protein. The effects of ovariectomy on the cholinergic modulation of excitatory synaptic transmission were also assessed by quantifying the effect of the acetylcholinesterase inhibitor eserine on excitatory postsynaptic field potentials in vitro. Evoked excitatory synaptic responses are reduced by activation of M_1_-like muscarinic receptors in the entorhinal cortex [30, 45], and a smaller eserine-induced reduction in synaptic responses could occur if cholinergic function is compromised by ovariectomy.

## Methods

### Subjects and surgery

Experiments were conducted according to the guidelines of the Canadian Council on Animal Care and were approved by the Concordia University Animal Research Ethics Committee (Permit Number: 30000253). Female Long-Evans rats (Charles River) were pair-housed under a 12-hour light-dark light cycle. Animals that received ovariectomies underwent surgery on PD63. Rats were anaesthetized with 3% isoflurane in O_2_ and ovaries were removed via a single 2 cm lumbar skin incision and bilateral tears in the abdominal musculature. Groups of animals received either a sham surgery in which only the skin incision was made, ovariectomy, or ovariectomy and immediate implantation of a subcutaneous capsule in the nape of the neck to maintain low circulating levels of 17β-estradiol (E2) [43]. Capsules were made using the materials and methods of Almey et al. (2013) which have resulted in serum E2 of 38 pg/ml 7 to 14 days following ovariectomy, and 29 pg/ml 21 days following ovariectomy [44]. Silastic tubing (1 cm-long; Dow Corning, I.D. 1.47 mm, O.D. 1.96 mm) sealed with silicone contained 8 mg of 5% cyclodextrin-encapsulated 17β-estradiol (Sigma Aldrich) in cholesterol (Bioshop Canada). Animals received postsurgical injections of buprenorphine (0.05 mg/kg, s.c.) every 12 hours for 72 hours.

### Protein quantification

#### Tissue preparation

Tissue was prepared for protein quantification 21 to 26 days following surgery. Subjects were anaesthetized with isoflurane and brains were rapidly removed and cooled (4°C) in oxygenated (95% O_2_ and 5% CO_2_) high sucrose ASCF solution containing, in mM, 2 KCl, 1.25 NaH_2_PO_4_, 7 MgSO_4_, 26 NaHCO_3_, 250 sucrose, 10 dextrose and 0.5 CaCl_2_. Horizontal slices (400 μm) were obtained using a vibratome (Leica, VT1200), and were then kept at 32°C for 30 min in normal ACSF containing 124 NaCl, 5 KCl, 1.25 NaH_2_PO_4_, 2 MgSO_4_, 2 CaCl_2_, 26 NaHCO_3_, and 10 dextrose. The medial and lateral entorhinal cortex were isolated through the dorso-vental extent of the brain [46, 47]. Tissue was placed in normal ACSF consisting (in mM) of 124 NaCl, 5 KCl, 1.25 NaH_2_PO_4_, 2 MgSO_4_, 2 CaCl_2_, 26 NaHCO_3_, and 10 dextrose saturated with 95% O_2_ and 5% CO_2_ at 32°C for 30 min. Tissue was then kept in normal ACSF at 22–24°C for 30 min.

#### Protein extraction and western blotting

Tissue was collected into microfuge tubes and snap-frozen in liquid nitrogen. Tissues were then disrupted in radioimmunoprecipitation assay homogenization buffer (50 mM Tris, pH 7.4, 0.1% SDS, 150 mM NaCl, 1.0% NP-40, 0.5% sodium deoxycholate, 1 mM EDTA, and 1 mM PMSF) using a tissue sonicator (QSonica, Q55). The quantity of protein in each sample was determined using BCA Protein Assay (Thermo Fisher, 23227) and an ELISA Fluorostar Analysis System plate reader. Bovine serum albumin (BSA) was used as the standard for protein quantification. Protein samples (20 to 40 μg) were resolved on Tris-glycine 8–10% SDS-PAGE gels. The resolved proteins were transferred from gels to nitrocellulose membrane (Bio-Rad, 1620112) and blocked for 1 hour in either 5% milk or 5% BSA, depending on the specific antibody, in Tris-buffered saline (TBS) containing 0.2% Tween-20 (TBST).

Primary antibodies including anti-choline acetylcholinesterase (1:1000; Abcam, AB183591), anti-vesicular acetylcholine transporter (1:2000; Abcam, AB235201), anti-cholinergic muscarinic receptor 1 (1:2000; Alomone labs, AMR-010), anti-vinculin (1:4000; Abcam, AB130007), anti-β-Actin (1:5000; Abcam, AB8226), were diluted in TBST containing either of 5% milk or 5% BSA and incubated overnight at 4°C. Membranes were then washed 3 times for 5 minutes each in TBST and incubated at room temperature with either peroxidase-conjugated goat anti-mouse secondary antibody (used at 1: 3000 for anti-vinculin, and 1: 5000 for anti-β-Actin; Millipore Sigma, AP124P) or peroxidase-conjugated goat anti-rabbit secondary antibody (used at 1: 5000 for anti-acetylcholinesterase, 1: 6000 for anti-vesicular acetylcholine transporter, and 1:4000 for anti-cholinergic muscarinic receptor 1; Millipore Sigma, AP132P) for 1–2 hours. Immunoreactivity was detected using ECL Western blotting substrate (Thermoscientific, 32106) and visualized using a CDP-STAR chemiluminescence system (Amersham hyperfilm ECL). Western blot data were compiled from at least six animals, and bands were quantified by densitometric analysis using Image-J software (version 1.41).

Results were analysed using GraphPad Prism software version 8.0.1 and analyzed with a mixed design Group (Intact, OVX, OVX+E) by Site (MEC vs LEC) analyses of variance (ANOVA) and Tukey comparisons. Antibody signals were normalized against the largest loading control immunoreactivity recorded in the medial or lateral entorhinal cortex of rats that received sham surgery. Bar graphs indicate the mean and standard error of the mean, normalized to the largest control value in percentage, and include values obtained from individual animals.

### Electrophysiological recordings

Recordings were obtained 7 to 17 days after ovariectomy on PD70 to PD80, and conducted during the dark phase of the light-dark cycle. Horizontal brain slices (400 μm-thick) containing the hippocampal and entorhinal regions were obtained and maintained for at least 30 min at 22–24°C prior to recordings. Slices were placed on a nylon net in a gas-fluid interface recording chamber (Fine Science Tools) and perfused at 2.0 ml/min with the upper surfaces exposed to humidified 95% O_2_, 5% CO_2_ atmosphere. Field excitatory postsynaptic potentials (fEPSPs) were recorded using borosilicate glass pipettes (1.0 mm OD; Sutter Model P97) filled with ACSF (4 to 6 MΩ). Electrodes were positioned 75 to 200 μm below the surface of the slice in layer I of the lateral entorhinal cortex, close to the layer II border, using a dissecting microscope (Leica, MS5). Bipolar stimulating electrodes made from tungsten electrodes (0.8–1 MΩ, FHC Inc.) were placed in the middle of layer I, 0.3 to 0.4 mm rostral to the recording electrode.

Changes in fEPSPs induced by the acetylcholinesterase inhibitor eserine hemisulfate (Sigma) were compared in slices obtained from intact rats and ovariectomized rats with and without E2 capsules. Constant current pulses (0.1 ms duration) were delivered every 30 seconds with a stimulus generator and stimulus isolation unit (WPI, A300 and A365), using a current that elicited fEPSPs approximately 65% of the maximal response (50–135 μA). Field EPSPs were amplified (DC-3 kHz; Axoclamp 2B, Molecular Devices) and digitized (20 kHz; Digidata 1322A) using Clampex 8.2 software (Molecular Devices). In each slice, after a baseline period of at least 10 min in which fEPSP amplitude varied less than 10%, 10 μM eserine was added to the ACSF for a period of 15 minutes. There was then a 40 min follow-up period in normal ACSF. Peak amplitudes of fEPSPs were measured using pClamp 8.2 software (Molecular Devices), and data for each slice were expressed as a percent of the average baseline fEPSP amplitude. The average fEPSP amplitude obtained during the baseline period, and 25–30 and 50–55 min after the onset of eserine, were assessed using a mixed design ANOVA with Group (Intact, OVX, OVX+E) and Time (baseline, 25–30 min, 50–55 min) as factors. Significant effects were investigated with post-hoc Tukey’s comparisons.

## Results

### Protein quantification

**Fig 1:**
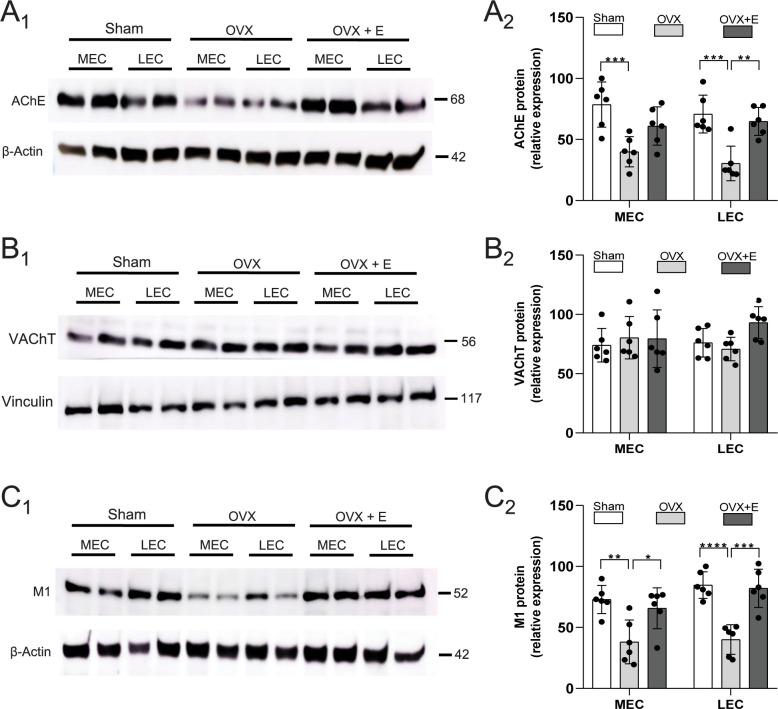
Ovariectomy results in reductions in proteins associated with cholinergic synaptic function in the entorhinal cortex. Lysates were obtained from the medial and lateral entorhinal cortex (MEC and LEC), in groups of animals that received either sham surgery (Sham), ovariectomy (OVX), or ovariectomy and a subdermal implant containing 17-β estradiol (E2; OVX+E). **A.** Representative immunoblots of acetylcholinesterase (AChE) and the β-actin loading control are shown (A_1_), and the bar graph shows relative expression of AChE protein (A_2_; n = 6 per group). Note that the reduction in AChE induced by ovariectomy is prevented by administration of E2. Asterisks indicate levels of statistical significance (*, *p* < 0.05; **, *p* < 0.01; ***, *p* < 0.001; ****, *p* < 0.0001). **B.** No significant changes were observed in immunoblots (B_1_) or normalized protein expression (B_2_) for the vesicular acetylcholine transporter (VAChT; n = 6 per group; vinculin was the loading control). **C.** Representative immunoblots (C_1_) and relative protein expression (C_2_) indicate that the reduction in M1 receptor protein induced by ovariectomy was prevented by administration of E2.

Western blot quantification was used to measure proteins related to cholinergic function, and to determine if changes in protein expression induced by ovariectomy could be blocked by chronic E2 administration (n = 6 per group). In comparison to tissue from animals that received sham surgery, ovariectomy resulted in marked reductions in AChE protein immunoexpression (F_2, 20_ = 16.54, p<0.0001) in both the medial (p = 0.003) and lateral entorhinal cortex (p = 0.002) (Fig 1A). Further, the replacement of E2 following ovariectomy prevented significant reductions in AChE in both the medial (p = 0.208) and lateral entorhinal cortex (p = 0.821). Differences in AChE in ovariectomized rats versus ovariectomized rats with E2 replacement were significant for the lateral (p = 0.007), but not medial entorhinal cortex (p = 0.113). Ovariectomy therefore causes a reduction in AChE protein in the entorhinal cortex that is prevented by chronic administration of E2.

Although the vesicular acetylcholine transporter (VAChT) is expressed broadly and serves as a marker for cholinergic axon terminals [48], we found that ovariectomy did not significantly affect expression of VAChT. Results showed no significant main effect of group (F_1, 30_ = 0.15, p = 0.706) on VAChT expression (Fig 1B).

Ovariectomy resulted in a strong reduction in M_1_ muscarinic receptor protein expression in both the medial and lateral entorhinal cortex in comparison to the sham control group (F_2,20_ = 27.22, p<0.0001; medial, p = 0.001; lateral, p<0.0001). Replacement of E2 prevented the reduction in M_1_ receptor protein. There was no significant difference in M_1_ receptor expression between control animals and ovariectomized animals that received E2 replacement (medial, p = 0.660; lateral, p = 0.946). Muscarinic M_1_ expression was also significantly reduced in ovariectomized animals in comparison with ovariectomized animals that received E2 replacement (medial, p = 0.009; lateral, p<0.001) (Fig 1C). Ovariectomy therefore causes reductions in M_1_ protein in both the medial and lateral entorhinal cortex that are prevented by chronic administration of E2.

### Effects of eserine on field EPSPs

**Fig 2:**
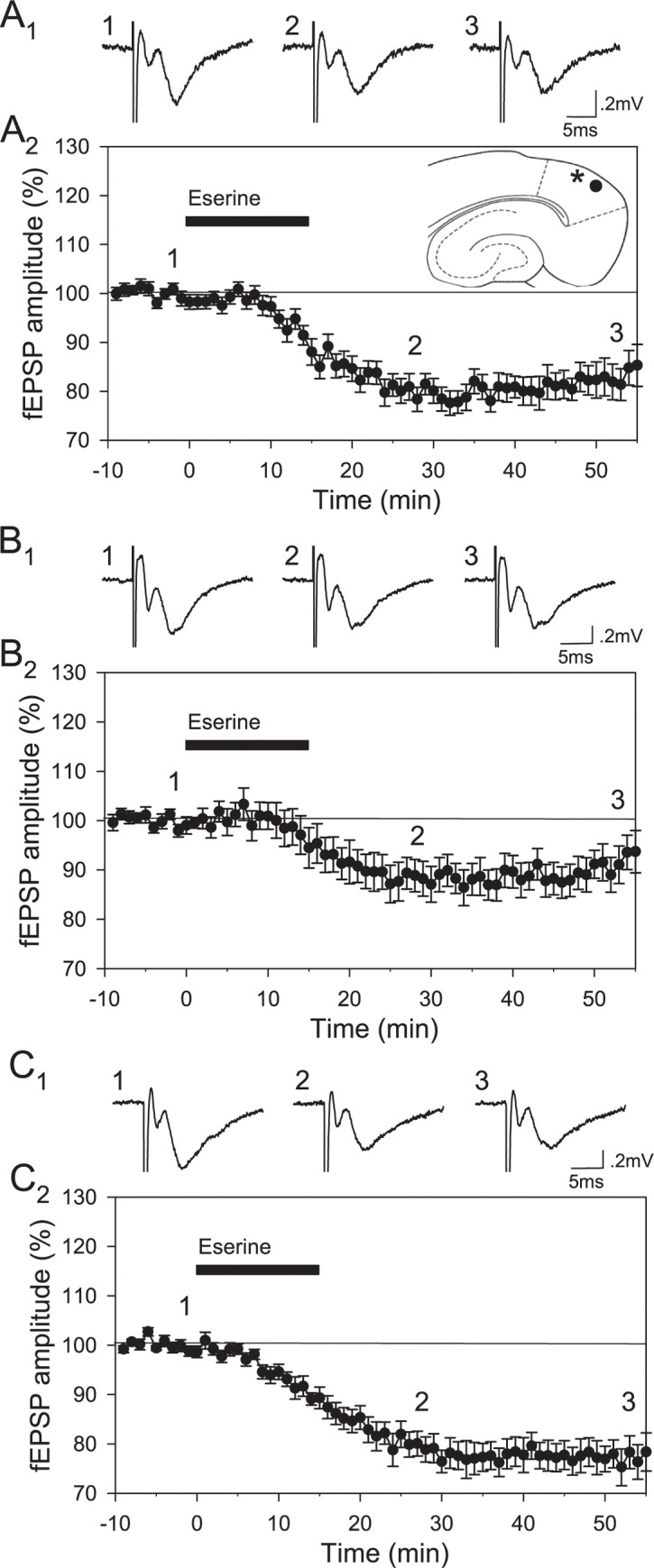
Ovariectomy reduces cholinergic modulation of excitatory synapses. The acetylcholinesterase inhibitor eserine differentially modulates field excitatory postsynaptic potentials (fEPSPs) in the lateral entorhinal cortex in brain slices obtained from intact rats (A), ovariectomized rats (B), and ovariectomized rats with E2 capsules (C). **A.** Representative averaged fEPSPs from a slice obtained from an intact rat (A_1_) are shown for the baseline period (trace 1), 28 min after onset of application of 10 μM eserine (trace 2), and at the end of the recording period (trace 3). Traces are averages of five consecutive responses. In slices from intact rats, mean fEPSP amplitude was reduced following application of eserine (black bar; n = 13) (A_2_). The inset diagram shows typical locations of the stimulating (*) and recording (circle) electrodes in horizontal slices of the lateral entorhinal cortex. Numbers indicate the times at which traces in A_1_ were obtained, and bars indicate ± one standard error of the mean. **B.** Representative averaged traces are shown for a slice obtained from an ovariectomized rat (B_1_), and changes in mean amplitude of fEPSPs in the group of slices are shown (B_2_; n = 10). **C.** Representative traces (C_1_) and averaged changes in mean fEPSP amplitude (C_2_, n = 10) are shown for a group of slices obtained from ovariectomized rats that received E2 replacement. Reductions in fEPSP amplitude induced by eserine were significantly smaller in ovariectomized rats versus either intact rats or ovariectomized rats that received E2 replacement, both 25–30 min and 50–55 min after eserine application (p <0.05).

Application of eserine resulted in reductions in the amplitude of field excitatory postsynaptic potentials (fEPSPs) that began about 10 min after the onset of eserine application, and were maximal after approximately 25 to 30 min during the wash period. In intact rats, EPSP amplitude was reduced to 79.1 ± 2.2% of baseline levels 25–30 min after the onset of the drug, and to 83.0 ± 3.2% at the end of the recording period (n = 13 slices from 7 rats); Fig 2A). The size of the reduction was smaller in ovariectomized animals (11.5 ± 2.9% versus 20.9 ± 2.2% in intact rats), and fEPSP amplitudes in slices from ovariectomized animals were at 88.1 ± 3.2% of baseline 25–30 min after onset of eserine, and 91.7 ± 3.5% of baseline at the end of the recording period (n = 10 slices from 6 rats; Fig 2B). Ovariectomized rats that received E2 replacement showed declines in fEPSP amplitude similar to intact rats, and fEPSP amplitudes were 78.9 ± 2.5% of baseline 25–30 min after onset of eserine, and 77.8 ± 3.1% of baseline at the end of the recording period (n = 10 slices from 5 rats; Fig 2C). An ANOVA showed a significant group by time interaction (F_4,60_ = 3.51, p = 0.023). Tukey’s comparisons indicated that, although reductions in fEPSPs did not differ between intact rats and ovariectomized rats that received E2 replacement (25–30 min, p = 0.997; 50–55 min, p = 0.296), slices from ovariectomized rats showed significantly smaller fEPSPs as compared to intact rats (25–30 min, p = 0.028; 50–55 min, p = 0.034), and as compared to ovariectomized rats with E2 replacement (25–30 min, p = 0.034; 50–55 min, p < 0.001). Therefore, the reduction in fEPSP amplitude induced by eserine was significantly smaller in slices from ovariectomized animals versus intact rats, and this effect was prevented by replacement of E2 following ovariectomy.

## Discussion

Basal forebrain cholinergic neurons impact cognitive processing by modulating neuronal excitability and synaptic transmission in both the hippocampus and entorhinal cortex [30, 40, 45]. Reductions in estrogens following ovariectomy are thought to impair cognitive processes in part by reducing the function of cholinergic afferents to the hippocampus [15, 39], but reduced cholinergic input to the entorhinal cortex is also likely to impact cognitive function. We have found that ovariectomy results in reductions in both acetylcholinesterase (AChE) and M_1_ receptor protein in the medial and lateral entorhinal cortex, and that these reductions are prevented by chronic replacement of 17β-estradiol (E2). Ovariectomy also reduced the modulatory effects of the acetylcholinesterase inhibitor eserine on excitatory synaptic responses in the entorhinal cortex in vitro. Eserine induced a smaller reduction in the amplitude of field EPSPs in slices from ovariectomized vs intact rats, indicating a reduced cholinergic suppression of excitatory synaptic transmission [30], and this effect was prevented by chronic replacement of E2 following ovariectomy. This suggests that reductions in E2 following ovariectomy result in a functional impairment of cholinergic transmission in the entorhinal cortex.

The cognitive changes following ovariectomy that have been attributed to reduced basal forebrain cholinergic input to the hippocampus may be due in part to reduced cholinergic function in the medial and lateral entorhinal cortex [39, 49]. The medial entorhinal cortex contributes to navigation and spatial processing and memory [50] and the lateral entorhinal cortex is involved in olfaction, object recognition, and memory for object location [51, 52]. We have shown that ovariectomy reduced AChE and M_1_ receptor protein in both regions, and this may affect both local processing and the activity of entorhinal projections to the hippocampus [53].

### Reductions in cholinergic synaptic proteins

The finding that ovariectomy reduced AChE expression in entorhinal tissue is consistent with studies that have found reductions in ChAT mRNA in basal forebrain cholinergic nuclei [54–56]. Ovariectomy also results in widespread reductions in cholinergic projections to the hippocampus, prefrontal cortex, and olfactory bulbs as reflected by reductions in ChAT and high affinity choline uptake [36, 57, 58]. We found that the reduction in AChE in the entorhinal cortex of ovariectomized animals was prevented by maintaining circulating E2 using a subcutaneous implant [44]. Similarly, E2 replacement prevents reductions in cholinergic staining in hippocampus [36, 57] and prefrontal cortex [58]. It is not clear why levels of the vesicular acetylcholine transporter (VAChT) remained stable [59] while levels of AChE were reduced by ovariectomy, but this suggests that ovariectomy resulted in a reduction in the function of cholinergic terminals without substantial loss of synaptic terminals or vesicular machinery.

Muscarinic M_1_ receptor protein was reduced in the entorhinal cortex by ovariectomy, and this reduction was prevented by replacement of E2. These findings are consistent with the concurrent decrease in AChE protein, and with studies demonstrating reduced cholinergic function following ovariectomy [36, 57, 58]. Ovariectomy decreases M_1_ receptor mRNA in the hippocampus [49] and has been found to reduce M_1_ and M_2_ receptor binding in basal forebrain and cortical projection areas including the entorhinal cortex, although this effect was not prevented by E2 supplementation [60]. In contrast, others have found that M_1_ to M_5_ receptor protein is increased 15 days following ovariectomy in the hippocampus and that these increases are prevented by E2 supplementation [61, 62]. An increase in muscarinic M_4_ receptors on glutamatergic terminals may also underlie reduced glutamate release in the hippocampus following ovariectomy [63]. Increases in muscarinic receptors following estrogen supplementation are also thought to contribute importantly to the cognitive benefits of estrogen supplementation [64]. Compared to their non-treated counterparts, postmenopausal women that receive hormone replacement therapy have increased plasma estradiol and higher muscarinic receptor densities in the striatum, hippocampus and frontal cortex [65]. Estrogen replacement following ovariectomy also results in an enhancement of hippocampal long-term synaptic potentiation that is dependent on muscarinic receptors [63]. Differences in the effects of ovariectomy on muscarinic receptors may be related to experimental variables including duration of ovariectomy and brain region examined.

### Reduced effect of eserine on EPSPs

Reductions in fEPSP amplitudes began approximately 10 minutes after the onset of eserine application and persisted for the duration of recordings. The delayed onset of the effect is likely due to time needed for drug concentration to rise in our high-volume interface recording chamber, and for eserine to increase acetylcholine availability by blocking degradation of endogenously released acetylcholine. The reduction in fEPSPs did not reverse entirely; this may be due to the high binding affinity of eserine [66], but eserine can also induce a long-term depression of synaptic responses in the CA1 region via a lasting reduction in glutamate release [67], and this may have contributed to the duration of the effect.

Electrophysiological results obtained here are consistent with a functional impairment of cholinergic transmission in the entorhinal cortex following ovariectomy. The acetylcholinesterase inhibitor eserine reduces the amplitude of fEPSPs, by prolonging the effects of acetylcholine which causes an M_1_-mediated reduction in glutamate release [30, 45] that is likely to affect both AMPA and NMDA receptor-mediated responses. Eserine had a reduced effect on field EPSPs in the lateral entorhinal cortex in slices from ovariectomized animals. Ovariectomy is also likely to have similar effects in the medial entorhinal cortex which also showed reductions in AChE and M_1_ receptor protein. The reduced effect of eserine could result from a reduction in endogenous release of acetylcholine, leading to smaller eserine-induced increases in acetylcholine concentration. Results are also consistent with the reduction in M_1_ receptors that we have observed, which may include reductions in M_1_ receptors on glutamate terminals.

## Supporting information

S1 Fig (PDF)
