# Supplementary material for: Ovariectomy reduces cholinergic modulation of excitatory synaptic transmission in the rat entorhinal cortex
Source: PLoS One. 2022 Aug 8;17(8):e0271131. doi: 10.1371/journal.pone.0271131 (PMC9359571; doi:10.1371/journal.pone.0271131)

Acetylcholinesterase (AChE) and β-Actin Loading Control (all samples loaded in gels (n=6))

Figure 1A$_1$

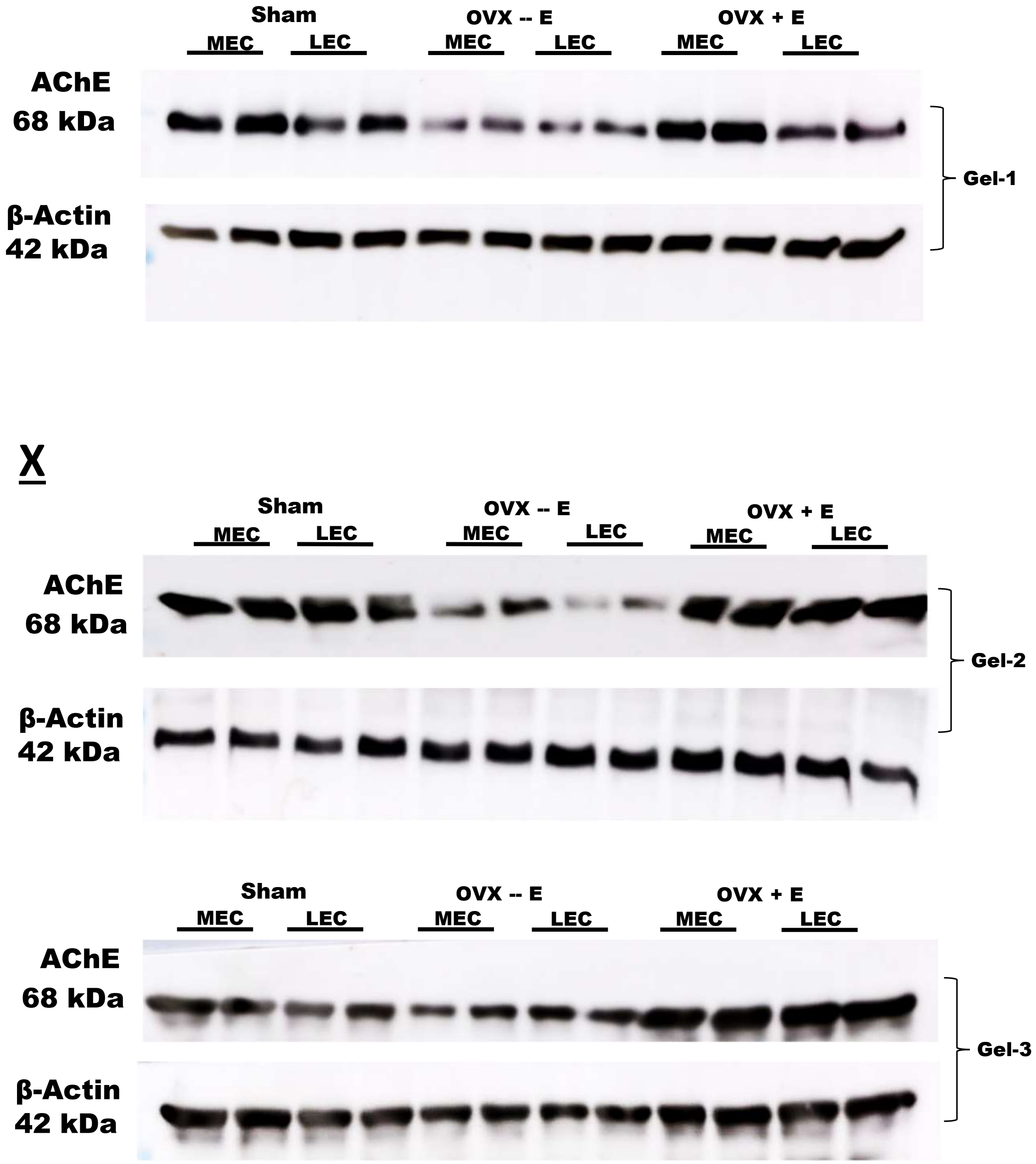

Vesicular acetylcholine transporter (VAChT) and Vinculin Loading Control (all samples loaded in gels (n=6))

Figure 1B$_1$

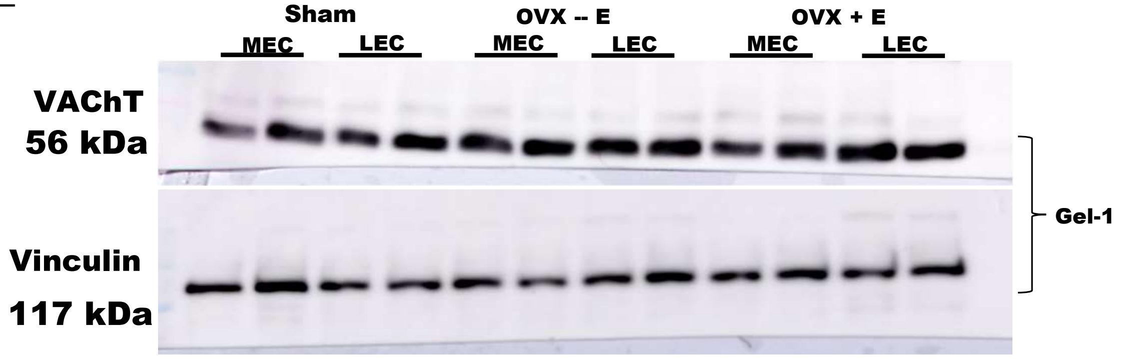

X

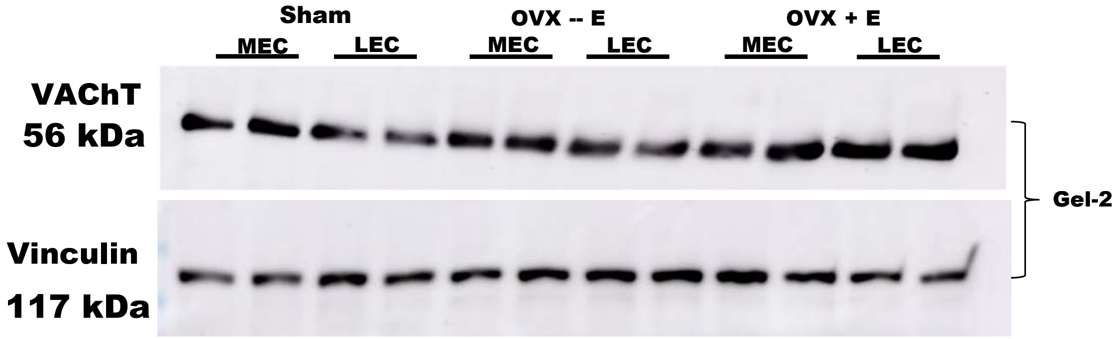

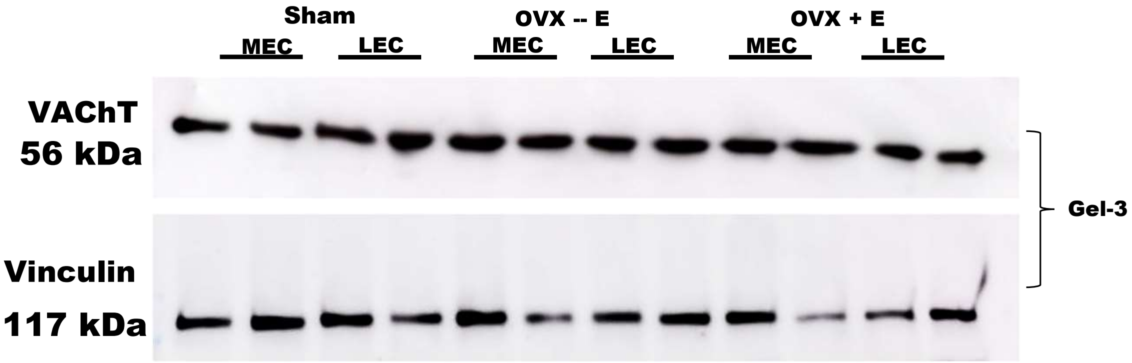

Muscarinic acetylcholine receptor (M1) and β-Actin Loading Control (all samples loaded in gels (n=6))

Figure 1C$_1$

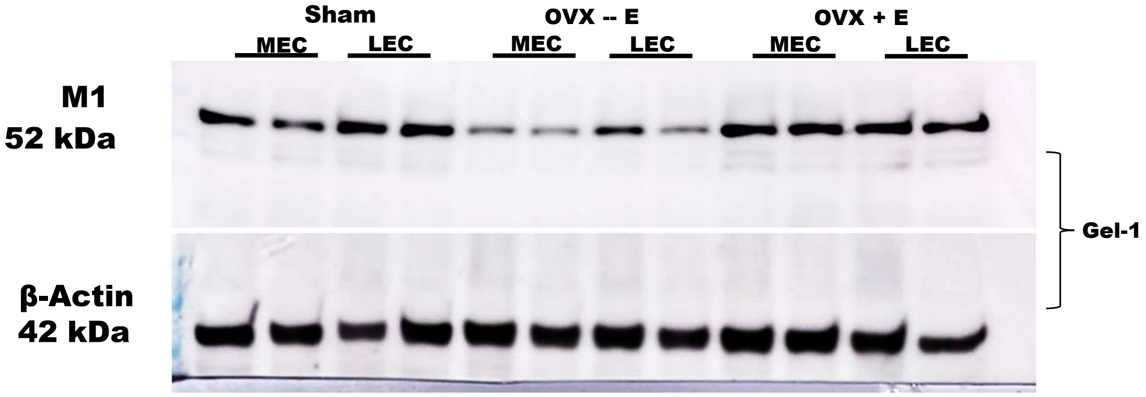

X

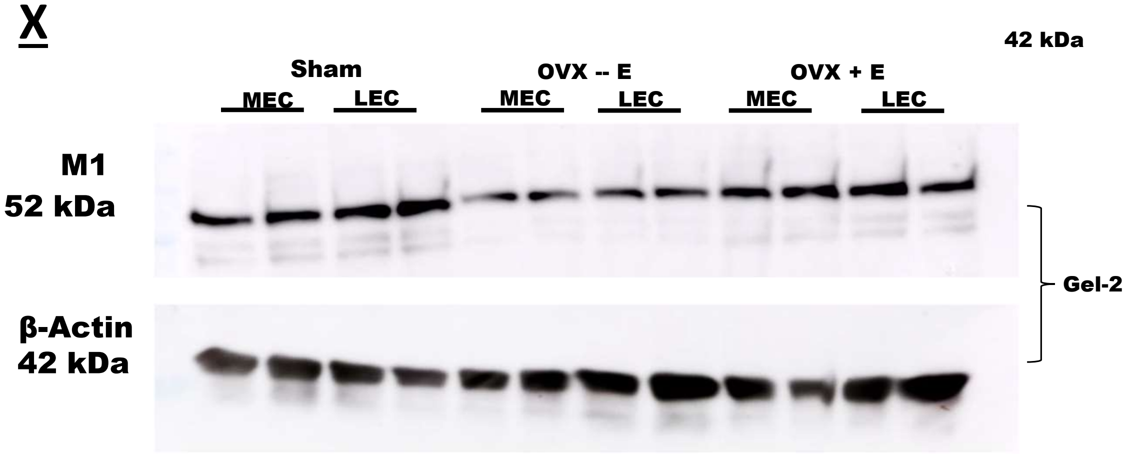

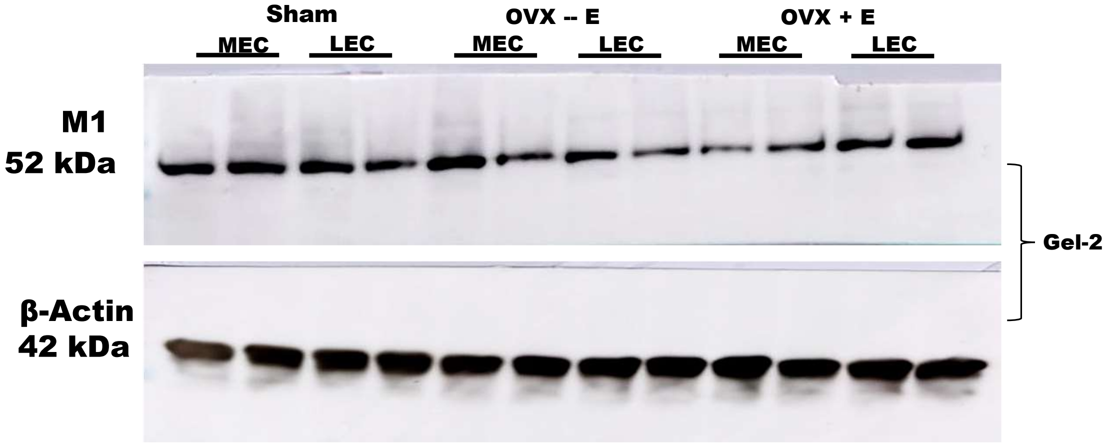

Supplement: S1 Fig — (PDF) [file pone.0271131.s001.pdf]
